# Aberrant Protein S‐Palmitoylation: A Novel Therapeutic Target in Alzheimer's Disease

**DOI:** 10.1002/alz70855_102201

**Published:** 2025-12-23

**Authors:** Claudio Grassi, Francesca Natale, Matteo Spinelli, Marco Rinaudo, Ida Nifo Sarrapochiello, Giuseppe Aceto, Daniela Puzzo, Salvatore Fusco

**Affiliations:** ^1^ Università Cattolica del Sacro Cuore, Rome, Rome, Italy; ^2^ Fondazione Policlinico Universitario A. Gemelli IRCCS, Rome, Italy; ^3^ Università Cattolica del Sacro Cuore, Rome, Italy; ^4^ University of Sassari, Sassari, Italy; ^5^ Oasi Research Institute‐IRCCS, Troina, Italy; ^6^ University of Catania, Catania, Italy

## Abstract

**Background:**

Protein post‐translational modifications are critical regulators of synaptic function, and their disruption is implicated in the pathogenesis of neurodegenerative diseases such as Alzheimer's disease (AD). Among these modifications, S‐palmitoylation, catalyzed by zinc finger DHHC domain‐containing (zDHHC) S‐acyltransferases, has been reported to modulate the localization and activity of proteins crucial for synaptic plasticity and amyloid‐β (Aβ) metabolism.

**Method:**

We analyzed zDHHC expression and protein S‐palmitoylation levels in hippocampi from 3×Tg‐AD mice and post‐mortem AD patient samples using western blotting and acyl‐biotin exchange assays. To evaluate the therapeutic potential of modulating S‐palmitoylation, we performed in vivo zDHHC inhibition through chronic intranasal administration of the S‐palmitoylation inhibitor 2‐bromopalmitate (2‐BP) or locked nucleic acid‐based antisense oligonucleotides targeting zDHHC7. Additionally, hippocampal zDHHC silencing was achieved using lentiviral vectors carrying short hairpin RNA. Palmitoyl‐proteome analysis was conducted on zDHHC7‐silenced hippocampi to identify specific targets involved in AD pathogenesis.

**Result:**

We observed a significant increase in zDHHC7 expression and S‐palmitoylation of synaptic proteins in the hippocampi of both 3×Tg‐AD mice and AD patients. Chronic intranasal administration of 2‐BP restored synaptic plasticity, reduced hippocampal Aβ deposition, improved cognitive function, and extended lifespan in both male and female 3×Tg‐AD mice. Similarly, hippocampal silencing of zDHHC7 prevented cognitive deficits in the same model. Palmitoyl‐proteome analysis identified several zDHHC7 targets potentially involved in neurodegeneration. Importantly, hippocampal protein S‐palmitoylation levels showed a significant inverse correlation with cognitive performance as measured by the Mini‐Mental State Examination in patients.

**Conclusion:**

These findings indicate that aberrant S‐palmitoylation plays a critical role in the synaptic dysfunction and cognitive impairments observed in AD. Targeting zDHHC enzymes represents a novel and promising therapeutic approach to address cognitive decline in AD patients by mitigating pathological S‐palmitoylation.

